# Nimotuzumab Concurrent with Gemcitabine as First-Line Treatment of Locally Advanced or Metastatic Pancreatic Adenocarcinoma

**DOI:** 10.1155/2023/1496072

**Published:** 2023-04-24

**Authors:** Yamirka Sánchez, Martha L. Concepción, Yohan Amador, Angel Piriz, René Rabassa, Ariel Leyva, Odalys Arguelles, Lisett Leblanch, Sheyla Moret, Gilberto Rivero, Ana L. Vasallo, Beatriz Martorell, Pedro P. Guerra, Ana R. Valls, Lisset Sánchez, Yaimarelis Saumell

**Affiliations:** ^1^Oncology Department, III Congreso Hospital, Pinar del Río 20100, Cuba; ^2^Oncology Department, Camilo Cienfuegos Hospital, Sancti Spíritus 60100, Cuba; ^3^Oncology Department, Faustino Pérez Hospital, Matanzas 40100, Cuba; ^4^Oncology Department, Agostinho Neto Hospital, Guantánamo 85100, Cuba; ^5^Oncology Department, Manuel Ascunce Domenech Hospital, Camagüey 70100, Cuba; ^6^Oncology Department, Vladimir Ilich Lenin Hospital, Holguín 80100, Cuba; ^7^Oncology Department, Antonio Luaces Iraola Hospital, Ciego de Ávila 65200, Cuba; ^8^Oncology Department, Carlos Manuel de Céspedes Hospital, Granma 85100, Cuba; ^9^Oncology Department, Medical and Surgical Research Center, Havana 11600, Cuba; ^10^Oncology Department, Ernesto Guevara de la Serna Hospital, Las Tunas 75100, Cuba; ^11^Oncology Department, Gustavo Aldereguía Lima Hospital, Cienfuegos 55100, Cuba; ^12^Oncology Department, Saturnino Lora Hospital, Santiago de Cuba 90500, Cuba; ^13^Clinical Trial Department, National Coordinating Center of Clinical Trials, Havana 11600, Cuba; ^14^Clinical Trial Department, Center of Molecular Immunology, Havana 11600, Cuba

## Abstract

**Background:**

Nimotuzumab exerts its antitumor effect (mainly antiproliferative, proapoptotic, and antiangiogenic) by blocking the epidermal growth factor receptor overexpressing between 30 and 95% in pancreatic tumors cells.

**Methods:**

A prospective, nonrandomized, uncontrolled, open-label, and multicenter clinical trial was conducted to evaluate the safety and effectiveness of nimotuzumab combined with gemcitabine as first-line treatment in unresectable locally advanced or metastatic pancreatic tumors in a real-world condition. Adverse events, their intensity, severity, and causality were determined using the Common Terminology Criteria for Adverse Events (CTCAE, version 4.0). Median overall survival, median progression-free survival, and 1- and 2-year survival rates were determined by using the Kaplan-Meier.

**Results:**

69 patients were included. The proportion of related serious adverse events was 1.2%. The most frequent adverse events were nausea (10%), anemia (8%), and abdominal pain (8%). Objective response was achieved in 18.5% of the patients and disease control in 43.1%. Patients with locally advanced disease achieved a median overall survival of 16.36 months (95% CI; 14.35-18.38); 1- and 2-year survival rates of 72.2 and 29.2 months, respectively; a median progression-free survival of 9.6 months (95% CI; 4.91-14.20); and a 1-year progression-free survival rate of 39%. Patients with metastatic disease achieved a median survival of 6.23 months (95% CI; 4.32-8.13); 1- and 2-year survival rates of 18.1 and 3.0 months, respectively; a median progression-free survival of 7.6 months (95% CI; 6.08-9.90); and 1- and 2-year PFS rates of 20.5 and 5.1 months, respectively.

**Conclusions:**

Nimotuzumab combined with gemcitabine represents a safe and effective first-line treatment option for patients with advanced pancreatic adenocarcinoma in real-world conditions. Survival benefits were increased in those patients who received 8 or more doses of nimotuzumab. This trial is registered with RPCEC00000245 in the Cuban Registry of Clinical Trials, part of the World Health Organization's International Clinical Trials Registry Platform (ICTRP).

## 1. Introduction

In 2020, pancreatic cancer (of 36 most common types of cancer) was ranked the 14^th^ place worldwide in terms of incidence and the 7^th^ place with respect the mortality [1]. Most patients with pancreatic cancer present advanced disease at diagnosis, and despite radical resection, the 5-year survival rate is only 10–25%. More than 80% of patients diagnosed with PC are not suitable for surgical treatment owing to local or distant metastasis [2].

The most used cytotoxic drug for the treatment of pancreatic adenocarcinoma has been gemcitabine, as monotherapy or combined with other treatments. The combination of gemcitabine plus nab-paclitaxel results more effective for locally advanced diseases than the rest of the combinations. The selection of the most appropriate regimen for each patient continues to depend on clinicopathologic stratification, and the development of appropriate biomarkers remains as a challenge [3]. There is not enough evidence to support which is the most appropriate regimen as a second line of chemotherapy after failure of the first line [4, 5]. In metastatic cancer, the preferred regimen is also the FOLFIRONOX regimen (5-fluorouracil, irinotecan, and oxaliplatin), although it results in a higher incidence of adverse events than gemcitabine alone [6]. The benefit of neoadjuvant therapy includes the elimination of micrometastasis and the reduction of the primary tumor, both factors associated with a decrease in the incidence of tumor recurrence [7]. However, phase III clinical trials are required to increase the quality of the evidence regarding its use [5].

The molecular characterization, the epigenomic, and the genomic profile of pancreatic adenocarcinoma have allowed a better understanding of the disease and have opened the doors to the study of other therapeutic approaches. The deeply immunosuppressive tumor microenvironment of pancreatic ductal adenocarcinoma, the dense stroma, the presence of inhibitory cytokines, few inhibitory T cells, and the low mutational tumor burden have played an important role in the failure of immunotherapy for the treatment of cancer of the pancreas [8]. Nevertheless, there are currently more than 150 clinical trials with multiple cytotoxic agents combined with new therapeutic agents that focus on the immune system, different therapeutic targets, the pancreatic stroma, and the tumor microenvironment, as adjuvant or neoadjuvant therapy for the treatment of pancreatic adenocarcinoma [5, 8]. Despite all efforts, survival at 5 years to date has only undergone modest changes and does not exceed 8% [5, 7], hence the importance of continuing to study new therapeutic alternatives.

The epidermal growth factor receptor (EGFR) is overexpressed in pancreatic tumor cells where it is believed to promote cell survival, proliferation, tissue invasion, and metastasis formation while inhibiting apoptosis [9]. In addition, overactivation of EGFR signalling contributes to downregulation of tumor cell immunogenicity through a decrease of HLA-I-dependent antigen presentation and upregulation of suppressive signals mediated by programmed death ligand 1 (PD-L1) and inhibitory cytokines, or by reprograming metabolic pathways after aerobic glycolysis upregulation [10].

Nimotuzumab is a humanized monoclonal antibody that mainly exerts its mechanism of action by competitively inhibiting the binding of the EGF ligand to the extracellular domain of the EGFR. The binding to EGFR leads to a further inhibition of the homodimerization or heterodimerization of this receptor and subsequent autophosphorylation of its tyrosine residues. The consequent inhibition of different canonical and noncanonical ligand-induced signal-transduction pathways results in suppression of tumor growth, inhibition of the mitogenic stimulation of malignant cells, and, therefore, their persistent proliferation and invasiveness. The processes of angiogenesis and metastasis are also inhibited, and apoptosis is activated. Nimotuzumab also elicits other immune responses such as the activation of natural killer (NK) cells and tumor cell lysis through antibody-dependent cellular cytotoxicity (ADCC). Furthermore, nimotuzumab activates adaptive immunity through tumor antigen- (TA-) specific CD8+ T cells and increases the expression of the human leukocyte antigen (HLA) class I-dependent antigen presentation molecule (known as signal 1) that allows induced T cells to recognize and kill EGFR+ tumor cells, reversing one EGFR-mediated mechanism of immune escape that can benefit tumors [10].

Nimotuzumab has been evaluated in clinical trials for the treatment of different types of cancer, obtaining evidence of benefits in terms of therapeutic effect and a broad safety profile [11–14].

Gao et al. found that nimotuzumab combined with gemcitabine-based chemoradiation promotes the enhancement of the cell cycle arrest, growth suppression, and apoptosis of EGFR-overexpressed pancreatic cancer cell lines PANC-1 [15]. In a clinical setting, a phase II study proved the safety and activity of nimotuzumab-200 mg as second-line monotherapy in patients with locally advanced or metastatic pancreatic cancer [16]. Another two clinical trials evaluated the synergistic effect of nimotuzumab-400 mg combined with gemcitabine-based regimen as first-line treatment in the same indication, a Germany phase II study [17] and a Chinese phase III study [18].

The present study was carried out to evaluate the safety and effect of nimotuzumab combined with gemcitabine in unresectable locally advanced or metastatic pancreatic tumors in a Cuban real-world conditions.

## 2. Materials and Methods

### 2.1. Study Design

A prospective, uncontrolled, nonrandomized, open, and multicenter study was conducted, to evaluate the safety and effectiveness of nimotuzumab, in combination with gemcitabine, in first-line treatment or recurrent patients with locally advanced and unresectable or metastatic pancreatic tumor in the real-world conditions. All included patients received a nimotuzumab fixed dose of 400 mg intravenously as a 30-minute infusion, once a week. The appearance of unacceptable toxicity were considered cause of definitive interruption, as well as the patient's refusal or death, and also, the discontinuation of nimotuzumab treatment for more than three weeks. Patients also received gemcitabine 1000 mg/m^2^ intravenously, 30 min infusion weekly, on days 1, 8, and 15, for 3 weeks followed by a 1-week rest. The treatment cycle was repeated until appearance of progression. Then, subsequent treatment options could include capecitabine or continuous infusion 5-FU.

### 2.2. Ethical Considerations

The study was conducted in agreement with the general principles adopted by the international community regarding biomedical research in human subjects, with current state regulations according to the requirements of the Cuban national regulatory agency, as well as in the Guide to Good Clinical Practices of the International Harmonization Conference (ICH E6). Besides, it was approved by the Cuban Minister of Public Health, the Institutional Ethics Committees of each hospital, and CECMED. The informed written consent was obtained from the patients before their inclusion in the investigation.

### 2.3. Eligible Patients

The main inclusion criteria are as follows: patients aged ≥ 18years who meet the diagnostic criteria and expressed written willingness to participate in the study by signing the informed consent, life expectancy equal to or greater than 3 months, clinical status according to ECOG criteria ≤ 2, and patients fit for chemotherapy and with normal renal and hepatic function according to laboratory parameters.

The main exclusion criteria are as follows: prior anticancer chemotherapy including adjuvant gemcitabine for pancreatic cancer, any investigational agent received concurrently or within the last 30 days, major surgery within the previous 3 weeks, previous or concurrent malignancy other than pancreatic cancer, uncontrolled ascites, or other clinically significant comorbidities.

### 2.4. Methods

The safety and effectiveness analyses were performed with all included patients who received at least one dose of nimotuzumab. The main variable was the proportion of patients with serious adverse events related to the administration of nimotuzumab (very probable, probable, and possible), and the corresponding 95% confidence interval was calculated. The frequency of patients with each adverse event was calculated, and the frequency distributions of each type of reported event. Severity and causality were determined by using the Common Terminology Criteria for Adverse Events (CTCAE, version 4.0).

The effectiveness analysis was performed independently for each stratum: locally advanced, metastatic, or recurrent patients, and overall survival (OS) and progression-free survival (PFS) were determined. The clinical response was evaluated as defined by the Response Evaluation Criteria in Solid Tumors (RECIST) version 1.0.

### 2.5. Statistical Analysis

Descriptive methods were used for patient's demographic and clinic characteristics, treatment exposure, and safety analysis. For the analysis of overall survival, median values and 95% confidence interval (CI) were estimated. OS and PFS were determined by the Kaplan-Meier methodology, and 1- and 2-year survival rates were estimated. For the clinical response, a point estimate and a 95% CI were performed for the proportion of patients with complete response (CR) and objective response (CR+PR). Statistical analysis was performed with SPSS program (version 21.0).

## 3. Results

**Figure 1:**
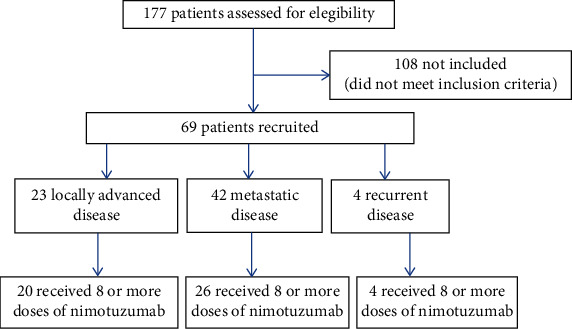
Distribution of patients evaluated in the study.

Between June 2017 and May 2020, 177 patients were evaluated. Of these, 69 were included in the study (Figure 1).

The largest number included were patients with metastatic disease. The data on the baseline characteristics of all the patients analyzed are summarized in Table 1.

### 3.1. Safety

Most of the study patients received 8 or more doses of nimotuzumab and at least 2 cycles of chemotherapy. Adverse events were reported in 49 patients (73.11%). A total of 319 adverse events of 83 different types were recorded. Treatment-related adverse events were recorded in 8.1% of patients. Severe AEs related to the treatment were registered in 1.5% of the patients, of these only four AEs (1.2%) related to the monoclonal (Table 2).

5.9% of the patients had treatment-related adverse events, and the proportion of patients with at least one serious adverse event related to the treatment was 1.5%. Most of the related AEs were mild and moderate and progressed towards recovery or improvement. No patient died from any adverse event associated with the use of the monoclonal antibody. The most frequent AEs were nausea (10%), anemia (8%), abdominal pain (8%), weakness (5%), and vomiting (5%). The frequency of related AE was 8.1%, and the proportion of serious AE related (very probable, probable, or possible) with the use of nimotuzumab was 1.2%.  Table 3 shows the treatment-related adverse events appears with nimotuzumab.

### 3.2. Survival Analysis

In the front-line setting, median overall survival of 9.0 months (95% CI; 8.36-15.11) was reached, and 1- and 2-year survival rates reached values of 38.8 and 7.6, respectively, while the overall mPFS was 8.03 ± 0.99 (6.08-9.90) months.

**Figure 2:**
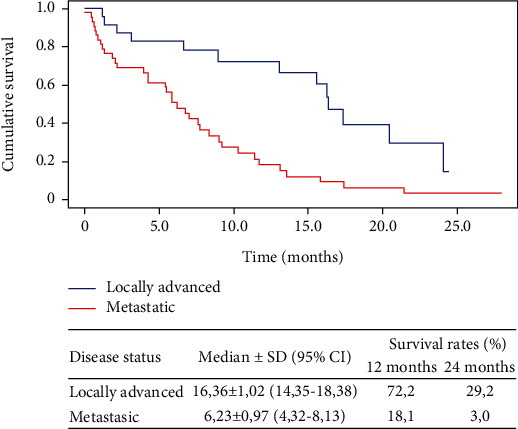
The Kaplan-Meier curves for overall survival in first-line treatment patients and according to disease status at inclusion. SD: standard deviation; CI: confidence interval.

In patients with locally advanced disease, a global median OS of 16.36 months (95% CI; 14.35-18.38) was reached, and 1- and 2-year survival rates reached values of 72.2 and 29.2, respectively. In patients with metastatic disease, a global median OS of 6.23 months (95% CI; 4.32-8.13) was reached, and 1- and 2-year survival rates reached values of 18.1 and 3.0, respectively (Figure 2).

**Figure 3:**
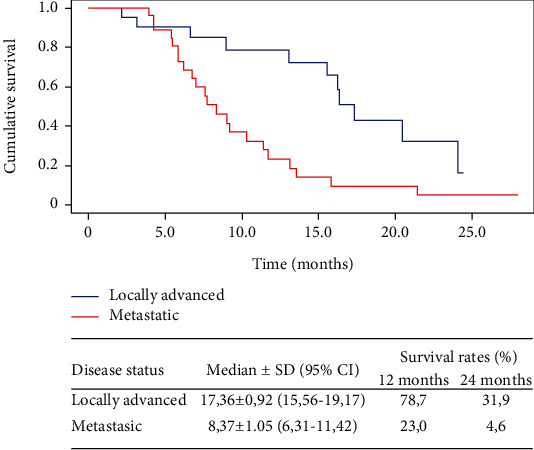
The Kaplan-Meier curves for overall survival in treatment patients with 8 or more doses of nimotuzumab and according to disease status at inclusion. SD: standard deviation; CI: confidence interval.

Figure 3 shows the results of overall survival for patients who received 8 or more doses of nimotuzumab. In 20 patients with locally advanced disease, the mOS increased to 17.4 months (IC95% 15.56-19.17), and 1- and 2-year survival rates reached values of 78.7 and 31.9, respectively. In 26 patients with metastatic disease, the mOS increased to 8.4 months (IC95% 6.31-11.42), and 1- and 2-year survival rates reached values of 23.0 and 4.6, respectively.

**Figure 4:**
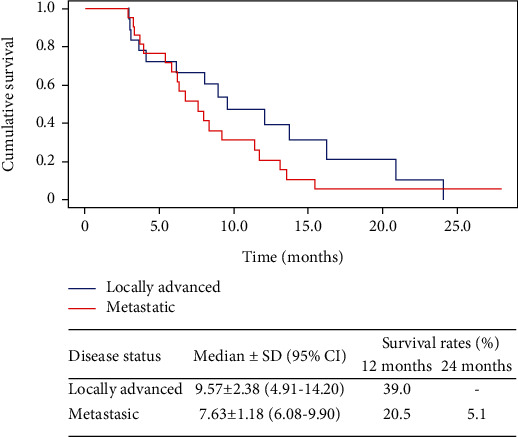
The Kaplan-Meier curves for progression-free survival in first-line treatment patients and according to disease status at inclusion. SD: standard deviation; CI: confidence interval.

In patients with locally advanced disease, a median PFS of 9.6 months (95% CI; 4.91-14.20) was reached, and the PFS rate at one year was 39%. No patient achieved the 2-year PFS rate. In 20 patients with locally advanced disease who received 8 or more doses of nimotuzumab, the mOS increased to 17.4 months (IC95% 15.56-19.17), and 1- and 2-year survival rates reached values of 78.7 and 31.9, respectively (Figure 4).

In patients with metastatic disease, a median PFS of 7.6 months was reached (95% CI; 6.08-9.90), and the PFS rates at one year and two years reached values of 20.5 and 5.1, respectively.

### 3.3. Clinical Response

The study obtained a 3.1% of complete remission and 15.4% of partial remission, which contributed to an objective response rate of 18.5%, and the disease control rate was achieved in 43.1% of the study patients (Table 4).

In patients who received 8 or more doses of nimotuzumab, the objective response increased to 26.1% and the disease control to 60.9%. In the stratum of patients with locally advanced disease, a 39.1% of objective response was obtained, and disease control was achieved in 60.9% of the patients. Those patients who received 8 or more doses of nimotuzumab reached a 45.0% of objective response and a 70.0% of disease control.

In patients with metastatic disease, a 7.14% of objective response was obtained (only partial remission), achieving control of the disease in 33.3% of the patients in this stratum. Patients who received 8 or more doses of nimotuzumab reached an 11.5% of objective response, and the disease control increased by 20%.

Of the four patients with recurrent disease upon inclusion, 3 achieved a partial response and one stable disease; thus, the control of the disease was achieved in all four.

## 4. Discussion

In the present study, with a mean follow-up time of 11.4 months, the very low toxicity profile of nimotuzumab was confirmed when administered in combination with gemcitabine in conditions of real-world practice.

The fundamental problem in terms of toxicity of the therapies that target the EGFR is the appearance of severe rash, infiltrated lymphocytes, folliculitis, and other adverse reactions that can cause in kidney cells and gastrointestinal mucosa. These reactions are caused by the interaction of these therapies with receptors found in other tissues of the body than the tumor [19, 20]. Nimotuzumab requires bivalent binding in conditions of high EGFR cell surface density for stable receptor docking and has a low affinity for normal tissue receptors, allowing it to selectively bind to EGFR-overexpressing tumor cells. The intermediate affinity of nimotuzumab results in an appropriate selectivity with an efficient localization to the tumors because their uptake by normal tissues with lower EGFR expression is very limited [21]. This accounts for the antitumor activity in the absence of severe adverse reactions seen with other anti-EGFR antibodies [20–22].

Table 5 summarizes the main results on safety and survival from clinical trials with combinations of anti-EGFR+chemotherapy as first-line treatment in patients with locally advanced or metastatic pancreatic adenocarcinoma.

In the phase III randomized clinical trial comparing the anti-EGFR monoclonal antibody cetuximab+gemcitabine versus gemcitabine as monotherapy, toxicity was significant to detriment of the monoclonal antibody. There was no significant difference between the groups when assessing overall survival, and the combination was less effective than gemcitabine alone; therefore, its use was not approved for the treatment of these patients [23].

Ko et al. studied patients with previously untreated locally advanced or metastatic pancreatic adenocarcinoma that were randomized to bevacizumab (10 mg/kg q2w) plus cetuximab (400/250 mg/m^2^ initial/weekly), either with (arm A) or without (arm B) gemcitabine (1000 mg/m^2^weekly 3x for 4 weeks). The primary study endpoint was progression-free survival (PFS). Sixty-one patients were randomized to arm A (*n* = 30) or arm B (*n* = 31). Median treatment duration was 9 weeks in arm A and 8 weeks in arm B (range, 2.0–40.4). Patients in arm A had median PFS and overall survival values of 3.55 months and 5.41 months, respectively, compared to 1.91 months and 4.17 months in arm B. The study closed early due to lack of sufficient efficacy in both treatment arms. Although both regimens were well tolerated, patients treated with gemcitabine experienced more grade 3–4 toxicities, including proteinuria and thromboembolic events. The combination of cetuximab and bevacizumab did not result in promising activity with or without gemcitabine [24].

The anti-EGFR tyrosine kinase inhibitor erlotinib was the first to show a slight increase in survival in combination with gemcitabine in the Phase III NCIC CTG PA.3 clinical trial that included 569 patients with advanced or metastatic pancreatic cancer, randomized to receive erlotinib+gemcitabine, or gemcitabine alone. A statistically significant benefit was obtained in favor of patients treated with the combination: in terms of global OS (HR, 0.82; *p* = 0.038) and PFS (HR, 0.77; *p* = 0.004). The mOS was 6.24 months, and the rate at one year was 23%, compared with 5.91 months and 17%, respectively, in the control group. In the group of patients treated with concurrent chemotherapy with erlotinib, rash and diarrhea, grades 1-2, and rash grade 2 were more frequent, although associated with better response and OS in this group [25].

The OSAG Phase III study that compared the combination of nimotuzumab and gemcitabine versus gemcitabine in patients with locally advanced or metastatic pancreatic cancer obtained a statistically significant clinical improvement in global OS and PFS with the combination of nimotuzumab and gemcitabine. In this study, data from 186 patients were analyzed: 93 in the nimotuzumab+gemcitabine group and 93 in the placebo+gemcitabine group. In patients treated with nimotuzumab, a 2-month advantage in overall survival was achieved, with a median overall survival of 8.6 months; survival rates at 12 and 18 months of 34% and 17%, respectively, in this group; and a statistically significant difference when compared with the control group (*p* = 0.03), which reached a median of 6.03 months and survival rates at 12 and 18 months of 19.2% and 9.0%, respectively. A 1.7-month PFS advantage was also obtained in patients treated with the combination, with a median PFS of 6.03 months and a 12-month PFS rate of 22.0% in this group and a difference statistically significant when compared to the control group (HR 0.68; *p* = 0.02), which reached a median of 5.1 months and survival rates at 12 and 18 months of 19.2% and 9.0%, respectively. The safety profile for the combination was favorable and comparable to that of the placebo group, with tremors, fatigue, and fever as the most frequent events (slightly) for the combination. Nimotuzumab was not associated with additional hematological adverse events, and only 15% of the patients experienced dermatological toxicity, mainly in grades 1-2 [17].

In another phase III clinical trial, a total of 92 Chinese patients were randomly assigned to the nimotuzumab-gemcitabine (*n* = 46) or placebo-gemcitabine groups (*n* = 46). In the full analysis set (FAS, *n* = 82), the mOS was significantly longer in the nimotuzumab-gemcitabine group (10.9 vs. 8.5 months, *p* = 0.025, HR 0.50, 95% CI (0.06 to 0.94). The 1-year survival rate was 43.6% in the nimotuzumab+gemcitabine group vs. 26.8% in the placebo+gemcitabine group and 13.9% vs. 2.7% at three years [18].

The safety and survival results for the nimotuzumab+gemcitabine combination also reflected in the abovementioned German and Chinese studies could be considered together with other clinical-demographic variables such as ECOG and age when thinking about a personalized therapy in a real-world condition.

Lima et al. evaluated the response to treatment and overall survival of 118 patients with advanced pancreatic adenocarcinoma in an observational and retrospective Cuban study. Patients were treated with nimotuzumab but combined with a GEMOX (gemcitabine+oxaliplatin) chemotherapy regimen. The median survival was 13.8 months (95% CI: 11.7-15.8). The second line of chemotherapy was administered to 42 patients, obtaining a median survival of 17.4 months (95% CI: 13.5-21.4). 51 grade 3-4 adverse events were reported, presented in 27 patients (22.9%). The most frequent adverse events were neuropathy (14.4%), neutropenia (10.2%), and thrombocytopenia (9.3%) [27].

The combination of leucovorin, gemcitabine, cisplatin, and 5FU (LGCF) combined with bevacizumab and cetuximab versus LGCF was compared in a phase III study in patients with locally advanced or metastatic pancreatic adenocarcinoma. The benefit was achieved to the detriment of immunotherapy, in mPFS (3.0 versus 9.0 months) and mOS (7.0 vs. 10.0 months) [26].

The phase III clinical trial of Imaoka et al. compared the combination of gemcitabine and S-1 (tegafur/gimeracil/oteracil), versus S-1 alone, and in a third group, gemcitabine alone, in patients older than 70 years with unresectable pancreatic cancer. It was found that there was no statistically significant benefit for the combination, superior to that of gemcitabine monotherapy. Median OS values of 10.2 months, 8.0 months, and 8.5 months, respectively, were reached [28].

When analyzing specifically survival results in locally advanced disease, the mOS of 16.4 months and the 1-year survival rate (72.2%) obtained in our study in this patients were higher than the value obtained by the patients in the Phase III OSAG study with the same disease status and treatment [17], which may be due to a synergistic effect between prolonged maintenance therapy with nimotuzumab (median treatment in patients with locally advanced disease was 42 administrations) and the effect of subsequent chemotherapy (median duration of chemotherapy was 6 cycles). In the OSAG study, in the group treated with nimotuzumab, the subgroup of 23 patients with locally advanced disease achieved a 1-year survival rate of 58.3%, without statistical significance when compared with the same subgroup in the placebo arm. However, there was a statistically significant difference when comparing the PFS rate at 12 months of 37.5% in this group, with that of the control group. The median duration of treatment in patients in this subgroup who received concurrent nimotuzumab therapy was 4 cycles (with a range of 1-21 days), which is equivalent to 16 administrations of nimotuzumab, while the observation time was 12 months. The authors considered that the survival benefit obtained was probably influenced by the administration of second-line chemotherapy (in more than 40% of the patients in each arm) [17].

In a 2016 randomized study that again compared gemcitabine alone versus gemcitabine and erlotinib, but only in patients with locally advanced disease, the median OS in the combination group was lower than that achieved in those treated with gemcitabine alone (11.9 versus 13.6 months, respectively) [29] and five months lower than the value obtained in our study.

The mOS obtained in patients with metastatic disease in the present study was five months lower than the value obtained by the FOLFIRINOX subgroup in the PRODIGE study and two months lower than the result obtained in the nab-paclitaxel+gemcitabine subgroup in the MPACT study. Also, the mOS obtained in the present study is one month higher than that achieved by the subgroup of 69 patients with metastatic disease treated with nimotuzumab in the OSAG study (17.2%) [17]. In patients who received more than eight doses of nimotuzumab, the benefit increased in five months (23%).

The Phase III study “MPACT” compared nab-paclitaxel+gemcitabine (431 patients) versus gemcitabine (430 patients) in metastatic patients. It obtained a median OS of 8.7 months in the group treated with the combination, OS rate of 35% at 12 months, and a median PFS of 5.5 months. An increase of 1.8 months in OS was obtained in the combination group compared to monotherapy [30].

In the Phase II-III trial “PRODIGE 4,” which compared gemcitabine with FOLFIRINOX versus gemcitabine in patients with metastatic pancreatic cancer and good performance status, an increase of 4.3 months in median survival was obtained in favor of the combination (11.1 vs. 6.8 months; *p* < .001). Similarly, a statistically significant benefit was obtained in PFS (6.4 vs. 3.3 months; *p* < 0.001) [6].

In our study, the overall objective response was lower than that achieved in other published studies. The overall disease control rate obtained in the present study was lower than that achieved in the group of patients treated with the same treatment scheme in the OSAG Phase III study [17], but it was increased by almost 20% in patients who received 8 or more doses of nimotuzumab. It may confirm the importance of long-term treatment with the monoclonal antibody.

The primary limitation of this study is that the number of subjects required to estimate the proportion of patients with serious adverse events related to the use of the product was not reached, and the sample size was small. Another limitation deals with the impossibility to perform any biomarker determination to identify subgroups of patients that could receive greater survival benefits. Nevertheless, there are evidence that pancreatic cancer cells with EGFR high expression were sensitive to nimotuzumab treatment in vivo [31] and results for EGFR-overexpression just reach statistical significance in the German clinical trial [17]. Regarding KRAS, the most commonly mutated gene in PDAC, Zhou et al. found that its status had no impact on antitumor efficacy of nimotuzumab in pancreatic cancer cells *in vivo* [31]. However, patients whose tumors harbored a KRAS wild-type experienced significantly better survival than those with KRAS mutations in two clinical trials [17, 18].

Immunotherapy and targeted therapy did not yield significative changes in pancreatic cancer, due to PDAC tumor immune microenvironment with lack of infiltrating T cells and low tumor mutational burden. Also, the biomarkers commonly used to predict immunotherapy efficacy in other tumors seem to be useless in PC [32]. However, immunotherapy remains a future breakthrough in the treatment of PDAC. An effective strategy to combat this “cold” tumor could be a multimodal immunotherapy combination of agents that target diverse immune-tumor interactions and multiple resistant mechanisms [33].

With the exception of erlotinib and nimotuzumab, all EGFR inhibitors failed in clinical trials in PDAC, indicating the presence of underlying molecular mechanisms that bestow intrinsic and acquired resistance to this group [34, 35]. Thus, only a subpopulation of PDAC patients benefit from EGFR inhibition (Table 5). In spite of this fact, nimotuzumab intermediate affinity seems to results in an adequate balance between antitumor potency and pharmacodynamics, conferring the monoclonal a safety advantage that allows its chronical administration whereas exhibiting a preferable toxicity profile among anti-EGF-R mAbs currently used in the clinical setting [20–22].

## 5. Conclusions

Nimotuzumab combined with gemcitabine as first-line treatment option represents a safe and useful alternative for the treatment of PDAC under real-world conditions. The administration of nimotuzumab as part of possible multimodal combination of agents that target a subset of cancer-associated fibroblasts or their secreted products (e.g., TGF-*β*), could be explored as an alternative to overcome the heterogeneity issues of pancreatic cancer. Also, with this purpose could be explored the possibility to develop next generation antibodies such as bi-specific antibodies or antibody-drug conjugates with this monoclonal. These treatment modalities may amplify an adaptive T cell immune response as well as offset mechanisms of resistance. Forthcoming researches will be done to validate the previous statement and to explore the crosstalk between EGFR canonical and noncanonical signalling pathways and their potential utility for additional therapeutic strategies with the monoclonal. Furthermore, finding surrogate biomarkers of response and evaluating the influence of tumor immune infiltration on the clinical outcome of nimotuzumab remains challenging.

## Figures and Tables

**Table 1:** Distribution of patients according to demographic and clinical variables.

Demographic and clinical characteristics		Locally advanced (*N* = 23)	Metastatic (*N* = 42)	Recurrent (*N* = 4)
Age (years)	Mean ± SD	61.2 ± 10.2	60.1 ± 10.9	60 ± 8.9
	Median ± QR	59 ± 15	59 ± 19	59 ± 17
	Min–Max	41-82	38-83	52-70
Weight (kg)	Mean ± SD	58.4 ± 11.3	58.9 ± 11.9	70.7 ± 12.8
	Median ± QR	58 ± 13	57.5 ± 17.5	71 ± 24.7
	Min–Max	42-86	29-85	56-85
Sex (no., %)	Female	8 (34.8)	15 (35.7)	1 (25.0)
	Male	15 (65.2)	27 (64.3)	3 (75.0)
Skin color (no., %)	White	14 (60.9)	30 (71.4)	1 (25.0)
	Black	4 (17.4)	9 (21.4)	1 (25.0)
	Mixed	5 (21.7)	3 (7.1)	2 (50.0)
ECOG (no., %)	0	10 (43.5)	11 (26.2)	—
	1	10 (43.5)	22 (52.4)	4 (100)
	2	3 (13.0)	9 (21.4)	—
Tumor location (no., %)	Head	15 (65.2)	19 (45.2)	3 (75)
	Body	2 (8.7)	4 (9.5)	—
	Tail	—	7 (16.7)	—
	Mixed	6 (26)	12 (28.6)	1 (25)

SD: standard deviation; QR: quartile range.

**Table 2:** General information on adverse events.

Categories by patient^1^ and adverse events (AEs)	Locally advanced (%)	Metastatic (%)	Recurrent (%)	Total (%)
Patients with an adverse event	18 (78.3)	27 (67.5)	4 (100)	49 (73.1)
Patients with TRAE	1 (4.35)	2 (5.0)	1 (25)	4 (5.9)
Patients with grade 3-4 TRAE	—	1 (2.5)	—	1 (1.5)
Adverse events	125 (39.2)	160 (50.2)	34 (10.7)	319 (100)
Treatment-related AE	11 (8.8)	14 (8.7)	1 (2.9)	26 (8.1)
Grade 3-4 treatment-related AE	—	4 (2.5)	—	4 (1.2)

^1^Subject can be included in more than one category. TRAE: treatment-related adverse events.

**Table 3:** List of treatment-related adverse events according to CTCAE^∗^ (version 4.0).

Treatment-related adverse event (TRAE)	No. of patients with the TRAE	Adverse events severity	
		Grade 1-2	Grade ≥ 3
Anorexia	1	1	—
Alkaline phosphatase increased	3	3	—
Alanine aminotransferase increased	1	1	—
Aspartate aminotransferase increased	1	1	—
Anemia	1	1	—
Glottis edema	1	—	1
Headache	4	3	1
Hematuria	1	1	—
Legs muscle weakness	1		1
Leukopenia	1	1	
Myalgia	4	4	—
Nauseas	5	5	—
Perioral cyanosis	1	1	
Rash	1	—	1

^∗^CTCAE: Common Terminology Criteria for Adverse Events.

**Table 4:** Clinical response for the first-line treatment stratum.

Response to treatment	Global population		
	Locally advanced *N* (%)	Metastatic *N* (%)	Total *N* (%)
Complete response (CR)	2 (8.7)	—	2 (3.08)
Partial response (PR)	7 (30.4)	3 (7.14)	10 (15.4)
Stable disease (SD)	5 (21.7)	11 (26.2)	16 (24.6)
Progressive disease (PD)	4 (17.4)	6 (14.3)	10 (15.4)
No objective evaluation (NE)^∗^	5 (21.7)	19 (45.2)	24 (36.9)
Objective response (OR)	9 (39.1)	3 (7.14)	12 (18.5)
95% confidence interval (CI)	26.0-73.9	3.2-37.8	15.5-7.7
Disease control (DC)	14 (60.9)	14 (33.3)	28 (43.1)
95% confidence interval (CI)	52.3-93.5	45.7-88.1	58.4-9.0
Total	23 (100)	42 (100)	65 (100)

^∗^Included patients who had either early death or symptomatic deterioration but no objective evaluation.

**Table 5:** Summary of the main results of clinical trials with anti-EGFR+chemotherapy as first-line treatment in patients with locally advanced or metastatic adenocarcinoma of the pancreas.

Study	Arms	mPFS (months)	mOS (months)	Clinical response (%)	Safety concerns	Reference
OSAG Phase IIb (Schultheis et al., 2017)	Nimotuzumab 400 mg+gem (*n* = 93) versus gem+placebo (*n* = 93)	5.1 vs. 3.4 (HR = 0.68; *p* = 0.0163)	8.6 vs. 6.0 (HR = 0.69; *p* = 0.0341)	CR: - PR: 8.6 vs. 8.6 SD: 54.8 vs. 43.0 DC: 63.0 vs. 52.0	The most frequent adverse events were fatigue (21.5% of patients, one patient grade 3), pyrexia (in 16.1%), chills (in 11.8%), and rash (in 15.1%, two patients grade 3)	[17]
NOTABLE Phase III (Qin et al., 2022)	Nimotuzumab 400 mg+gem (*n* = 46) versus gem+placebo (*n* = 46)	4.2 vs. 3.6 (HR, 0.56; 95% CI, 0.12-0.99; *p* = 0.013)	10.9 vs. 8.5, (HR, 0.50; 95% CI, 0.06-0.94; *p* = 0.025)	No statistical difference in the ORR between the two groups (*p* > 0.05)	The most common grade 3 treatment-related AEs in the nim+gem group were neutropenia (11.1%), leukopenia (8.9%), and thrombocytopenia (6.7%). No grade 4 treatment-related AEs	[18]
Phase III (Phillip et al., 2010)	Cetuximab+gem (*n* = 224) versus gem (*n* = 202)	3.4 vs. 3.0 (HR = 1.07; 95% CI, 0.93 - 0.24; *p* = 0.18)	6.3 vs. 5.9 (HR = 1.06; 95% CI, 0.91-1.23; *p* = 0.19)	CR: - PR: 8.0 vs. 7.0 SD: 37 vs. 30 DC: 45.0 vs. 37.0 The ORR was similar in both arms of the study (*p* = 0.59)	Patients receiving cetuximab+gem: 16% of patients with grade 4-5 toxicities; 7 with grade 5 toxicities; cetuximab was associated with an increased frequency of allergic reactions and skin toxicities including acne and rash. 48% patients with grade 2-3 skin toxicities	[23]
Phase II (Ko et al., 2012)	Cetuximab+bevacizumab+gem (*n* = 30) versus cetuximab+bevacizumab (*n* = 31)	3.55 vs. 1.91	5.41 vs. 4.17	CR: 3.4 vs. 0.0 PR: 10.3 vs. 3.4 SD: 31.0 vs. 24.1 DC: 44.8 vs. 27.6	Patients treated with gemcitabine experienced more grade 3–4 toxicities, including proteinuria and thromboembolic events. The study closed early due to lack of sufficient efficacy in both treatment arms	[24]
NCIC CTG PA.3 Phase III (Moore et al., 2007)	Erlotinib+gem (*n* = 285) versus gem+placebo (*n* = 284)	3.75 vs. 3.55 (HR, 0.77; 95% CI, 0.64-0.92; *p* = 0.004)	6.24 vs. 5.91 (HR, 0.82; 95% CI, 0.69-0.99; *p* = 0.038)	CR+PR: 8.6 vs. 8.0 SD: 48.9 vs. 41.2 DC: 57.5 vs. 49.2 (*p* = 0.07)	Patients receiving erlotinib and Gem: higher frequencies of grade 1-2 rash, diarrhea, infection, and stomatitis. Seven patients had an interstitial lung disease- (ILD-) like syndrome possibly related to therapy	[25]
Phase II (Tai et al., 2016)	Cetuximab+bevacizumab+LGCF (*n* = 31) versus LGCF (*n* = 28)	9.0 vs. 3.0 (*p* < 0.0001)	10.0 vs. 7.0 (*p* = 0.004)	N/A	Targeted treatment group had a higher frequency of severe grade 3 nausea and vomiting than the conventional treatment group (74.2% vs. 7.1%, respectively)	[26]

CI: confidence interval; HR: hazard ratio; N/A: not available; ORR: objective response rate; OS: overall survival; PFS: progression-free survival; Gem: gemcitabine; LGCF: leucovorin, gemcitabine, cisplatin, and 5FU.

## Data Availability

The data sets used and/or analyzed during the current study are available from the corresponding author upon reasonable request.
